# Regular exercise and creatine supplementation prevent chronic mild stress-induced decrease in hippocampal neurogenesis via Wnt/GSK3β/β-catenin pathway

**DOI:** 10.20463/jenb.2018.0009

**Published:** 2018-06-30

**Authors:** Yea-Hyun Leem, Morimasa Kato, Hyukki Chang

**Affiliations:** 1 Department of Human Movement Science, Seoul Women’s University, Seoul Republic of Korea; 2 Department of Health and Nutrition, Yonezawa Nutrition University of Yamagata Prefecture, Yonezawa Japan

**Keywords:** Chronic mild stress, depression, regular exercise, hippocampal neurogenesis, Wnt/GSK3β/β-catenin pathway

## Abstract

**[Purpose]:**

Chronic stress can lead to mood-related psychomotor behaviors such as despair. Decreased hippocampal neurogenesis has been observed in patients with depression and in animal models of depression. Exercise enhances the population of the new born cells in the dentate gyrus (DG). A few studies have demonstrated that creatine has antidepressant effects in humans. However, the mechanism underpinning these effects is poorly understood. Therefore, we examined whether regular exercise and/or creatine was closely associated with the activity of the Wnt/GSK3β/β-catenin pathway in the hippocampal DG.

**[Methods]:**

Mice were subjected to 4 weeks of chronic mild stress starting a week prior to the start of a 4-week protocol of treadmill running and creatine supplementation. Tail suspension (TST) and forced swimming tests (FST) were carried out 2 days after the final treadmill running session. Immunohistochemical and western blot analyses were conducted to evaluate hippocampal neurogenesis, GSK3β activity, and nuclear β-catenin protein levels in the DG. Furthermore, Wnt signaling antagonism in the DG using stereotaxic injection was performed.

**[Results]:**

Chronic mild stress-induced increase in immobility in the TST and FST were restored by treadmill running and/or creatine supplementation. The number of Ki-67+ and doublecortin (DCX)+ cells were decreased by chronic stress, and this decline was reversed by the exercise and supplement regimen, along with the changes in GSK3β activity and nuclear β-catenin protein levels in the DG. Local antagonism of DG Wnt signaling caused an increase in immobility even 5 days after injection with C59.

**[Conclusion]:**

Regular exercise combined with creatine supplementation had a greater effect on hippocampal neurogenesis via the Wnt/GSK3β/β-catenin pathway activation compared with each treatment in chronic mild stress-induced behavioral depression.

## INTRODUCTION

Chronic stress is a risk factor for developing many affective disorders, such as depression, and has a high prevalence worldwide. Repeated or chronic exposure to stressful stimuli can impair the negative feedback regulation of the hypothalamic-pituitary-adrenal (HPA) axis, damaging the affected structures in the brain. Among brain areas, the hippocampus is particularly susceptible to stress^1,2^. Many studies have demonstrated that chronic stress causes hippocampal atrophy, reduces neurotrophin levels, such as brain-derived neurotrophic factor (BDNF), and decreases neurogenesis^3-5^. Hippocampal neurogenesis is well-known to be related to depressive symptoms, with a chronic stress-provoked decrease in the number of new born cells was reversed by prolonged antidepressant treatment^6-8^. Furthermore, neurogenesis was attenuated in a human fetal hippocampal progenitor cell treated with cortisol^9^. Thus, adult hippocampal neurogenesis is often used to evaluate antidepressant action because of the requirement of hippocampal neurogenesis for the behavioral effects of antidepressants.

Wnt signaling was first identified for its role in oncogenic effects. Since then, some studies have suggested that Wnt signaling contributes to adult brain function, after its dysregulation was implicated in mood-related behaviors such as depression and memory-related neurodegenerative pathways^10-12^. Furthermore, Wnt signaling was profoundly activated, along with an aberrant increase in hippocampal neurogenesis in a model of temporal lobe epilepsy^13,14^.

Creatine is an energy source that contributes to the improvement of muscle power and sports performance. Regarding brain health, creatine supplementation was reported to affect cognitive and emotional function^15^. Creatine intake attenuated depressive symptoms in experiments with animal and human subjects^16-18^. However, the molecular mechanism underlying the antidepressant effects of creatine remains elusive.

Exercise is well-established in improving brain health, including cognitive, mood-, and emotion-related functions. In particular, exercise is used as an effective strategy for stress management and treating depression, as well as its beneficial effects on body systems that can ameliorate the side effects of antidepressants side effects, such as nausea, increased appetite and weight gain, fatigue and drowsiness, sleep disturbance, suicide, and anxiety^19^.

Despite of the antidepressant action of exercise and creatine supplementation, the functional role of Wnt signaling in chronic stress-induced impaired hippocampal neurogenesis is yet to be explored and whether exercise and creatine supplementation affects this signaling in the hippocampal DG area in chronic stressed conditions is still unknown. Furthermore, if exercise combined with creatine treatment has a greater effect on the chronic stress signal pathway, the antidepressant role of exercise and creatine may be more greatly potentiated. Herein, we demonstrate that the chronic stress-induced defects of canonical Wnt signaling and hippocampal neurogenesis, as well as behavioral depression, were alleviated by regular exercise and/or creatine supplementation.

## METHODS

### Experimental mice

Male 7-week-old C57BL/6 mice were obtained from Daehan Biolink, Co., Ltd. (Eumsung, Chungbuk, Korea) and housed in clear plastic cages under specific pathogen-free conditions and a 12:12-h light-dark cycle (lights on at 08:00 and off at 20:00). The mice had free access to standard irradiated chow (Purina Mills, Seoul, Korea). The Animal Care and Use Committee of Seoul Women’s University approved all experimental procedures involving animals.

### Experimental design

To identify the antidepressant effects of creatine intake and/or regular exercise (each group N = 12; five groups: Control, CON; Stress-Control, ST-CON; Stress-Exercise, ST-Ex; Stress-Creatine, ST-Cr; Stress-Exercise+Creatine, ST-Ex+Cr), we applied chronic mild stress (CMS) and then performed a tail suspension (TST) and forced swim test (FST) 2 days later^18^. The CMS consisted of three different and sequential stress situations as follows: 1) inclining the cage by 20° from the horizontal for 48 h; 2) wetting their chip bedding with 200 ml of water for 24 h; 3) agitating the cages using a rotatory shaker at 180 rpm for 24 h (normal cage for 24 h between each situation). We repeated these stress situations for the mice for a period of 4 weeks. The creatine and exercise treatments were performed for 4 weeks commencing 1 week after CMS was first applied. The exercise groups (ST-Ex and ST-Ex+Cr) utilized a treadmill running for 50 min (5 m/min for 10 min, 8 m/min for 30 min, 5 m/min for 10 min, 0% grade). Exercise was conducted as often as 5 days per week at 18:00–20:00. Treadmill speed was increased gradually so as not to increase the stress on the experimental animals. For creatine supplementation, the creatine dose was created by mixing a pellet of creatine monohydrate (Sigma chemical; 0 kcal/g) corresponding to a 4% intake-volume of the normal diet to produce a pellet^18^. To identify the intake volume of creatine and chow, we calculated the relevant amounts daily for all groups.

### Behavioral assessment

For the FST (each group N =12), mice were individually placed in an acrylic cylinder (100-mm diameter × 250- mm height) containing water (24 ± 1°C) to a depth of 17 cm. All mice were exposed to a 15-min pre-test on day 1. The test was conducted 24 h later and the experiment was recorded using a video camera. For the TST (each group N = 12), mice were suspended in the air using an acrylic box 350 mm (height) ´ 350 mm (length) ´ 350 mm (width) with a hook (JEUNG DO Bio & Plant Co. LTD, Seoul, Korea) fixed with adhesive tape wrapped around the tail of the mouse at least 30 cm from the base. Each mouse was suspended by its tail for 6 min, and the immobility time was measured during the last 5 min, with the first minute ignored to allow for habituation.

### Preparation of nuclear fraction and western blot analysis

The dentate gyrus (DG) from mice (each group, N = 4) was isolated using tissue punches (1.25-mm diameter) from the bregma -1.70 mm to -2.54 mm. Nuclear proteins were prepared with a Subcellular Proteome Extraction Kit (Calbiochem). In brief, tissue samples were homogenized with 1 mL of extraction buffer I (20 mM HEPES [pH 7.9], 20 mM KCl, and 30% sucrose) and 5 μL of protease inhibitor cocktail (0.5 mM PMSF, 100 μg/ml aprotinin, 5 μg/mL leupeptin, and 5 μg/mL pepstatin; Calbiochem) and then centrifuged at 3,000 rpm for 10 min. The obtained pellets were agitated with 1 mL extraction buffer II (20 mM HEPES [pH 7.9], 20 mM KCl, 30% sucrose, and 0.5% NP-40) and 5 μL of protease inhibitor cocktail for 30 min. After centrifugation at 7,000 rpm for 10 min, the pellets were agitated with 0.5 mL of extraction buffer III (20 mM HEPES [pH 7.9], 100 mM NaCl, 20% glycerol, and 1 mM DTT), 1.5 μL of benzonase (350 U), and 5 μL of protease inhibitor cocktail. The final nuclear supernatant was obtained by centrifugation at 9,000 rpm for 10 min. Protein samples were electrophoretically separated on 10% polyacrylamide gels, transferred to nitrocellulose membranes (Amersham Bioscience, Buckinghamshire, UK), and incubated overnight with primary antibody in a blocking buffer at room temperature (22-24℃). The next day, they were washed in a washing buffer and incubated with horseradish peroxidase-conjugated secondary antibody for 2 h at room temperature. The optical density of each band was measured using Image J (NIH Image Engineering, Bethesda, MD). Anti-β-catenin (1:500) and anti-Lamin B1 (1:2,000) antibodies were obtained from Abcam (Cambridge, MA, USA).

### Immunohistochemical analyses

The anesthetized mice were perfused with 100 mM PBS (pH 7.4), followed by cold 4% paraformaldehyde in PBS. Every fifth section was taken from the region between bregma -1.82 mm and -2.18 mm. Free-floating sections were incubated with 0.3% H2O2, permeabilized with 0.3% Triton X-100, and nonspecific protein binding was blocked by incubation with 3% normal goat serum. Sections were incubated overnight at 4°C with anti-Ki-67 (1:1,000; each group N = 7), anti-doublecortin (1:2,000; each group N = 7), and anti-pGSK3β (Ser9, 1:500; each group N = 6) primary antibodies (Abcam, Cambridge, MA, USA; rabbit polyclonal) and subsequently with biotinylated secondary antibodies (Vector Laboratories; Burlingame, CA, USA, 1:200, respectively). They were then visualized using the ABC method (ABC Elite kit, Vector Laboratories; Burlingame, CA, USA). The sections were mounted and assessed in digital images (captured at 100× magnification) using Image J (NIH Image Engineering, Bethesda, MD).

### Stereotaxic injection

For the Wnt antagonism experiment, mice were anesthetized with 250 mg/kg body weight of tribromoethanol (each group N = 6). One μL of 150 ng C59 (30 μM; Tocris Bioscience) dissolved in 0.9% saline or a saline-only solution were stereotaxically injected into the bilateral DG (AP, -1.50 mm; ML, ±2.18 mm; DV, -1.85 mm). FST tests was performed 1 and 5 days after the end of drug injection.

### Statistical analysis

One-way analysis of variance and independent t-tests were performed using SPSS (SPSS for Windows, version 18.0; IBM Corporation, Armonk, NY, USA) to assess for significance. Post hoc comparisons were made using Newman- Keuls tests. All values are reported as mean ± standard error of the mean. Statistical significance was set at *p* < 0.05.

## RESULTS

### Chronic mild stress-induced increase in immobile time in TST and FST was reversed by treadmill running and/or creatine supplement

**Fig. 1.**
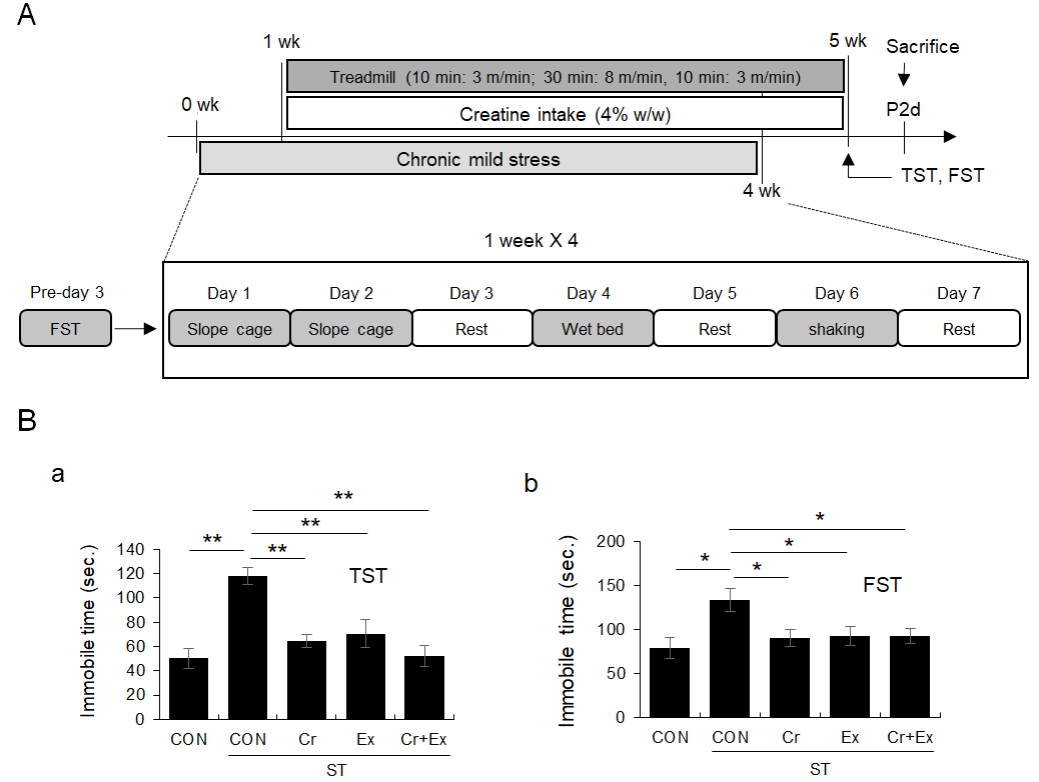
Regular exercise and/or creatine supplementation protected against chronic mild stress-induced increase in immobility during tail suspension test and forced stress test. A. Experimental design. B. Quantitative analysis for TST (a) and FST (b). Data are presented as the means ± SE (each group N = 12). * and ** denote differences at p < 0.05 and p < 0.01, respectively.

A 4-week chronic mild stress protocol increased immobility in the TST and FST, which was reversed by treadmill running for 4 weeks and creatine supplementation (Fig. 1B; for TST: F4, 55 = 9.63, *p* < 0.01; for FST: F4, 55 = 3.22, *p* < 0.05).

### Chronic stress-induced decrease in number of Ki- 76- and doublecortin (DCX)-positive cells in the DG was reversed by treadmill running and/or creatine supplement

**Fig. 2.**
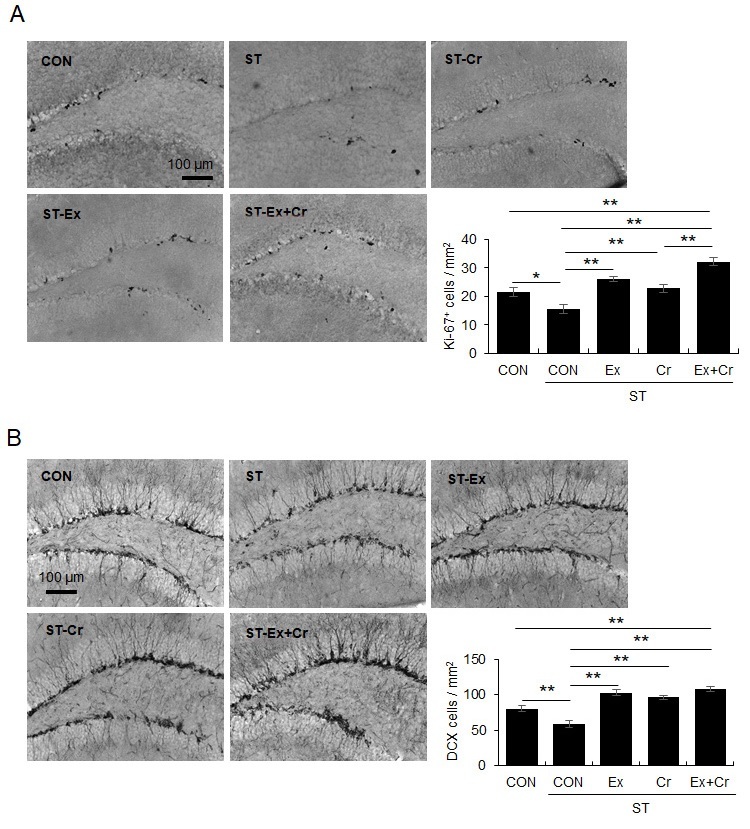
Regular exercise and/or creatine supplementation prevented chronic mild stress-induced decrease in the number of Ki-67- and DCX-positive cells in the DG. A. Photomicrograph showing immunohistochemical analysis for Ki-67. B. Photomicrograph showing immunohistochemical analysis for DCX. Data are presented as the means ± SE (each group N = 4). * and ** denote differences at p < 0.05 and p < 0.01, respectively.

A 4-week chronic mild stress protocol reduced the number of Ki-67+ and DCX+ cells, a reduction reversed by treadmill running for 4 weeks and creatine supplementation (Fig. 2A: for Ki-67, F4, 30 = 18.32, *p* < 0.01; Fig. 2B: for DCX, F4, 30 = 21.87, *p* < 0.01).

### Chronic stress-induced decrease in number of pGSK3β-positive cells and nuclear β-catenin levels of DG were reversed by treadmill running and/or creatine supplement, as well as the enhanced immobility by the local blockage of Wnt agonist, C59

**Fig. 3.**
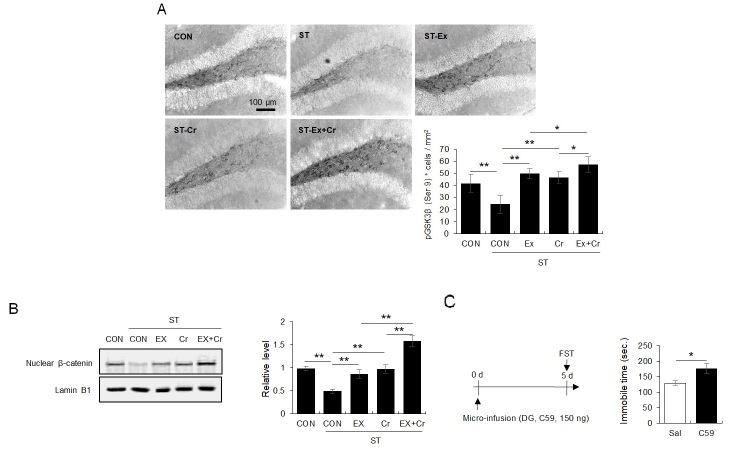
Regular exercise and/or creatine supplementation reversed the hcronic mild stress-induced decrease in Wnt/GSK3β/β-catenin signaling in the DG. A. Photomicrograph showing immunohistochemical analysis for pGSK3β (each group N = 6). B. Photomicrograph showing immunoblot analysis for nuclear β-catenin in the DG (each group N = 4). C. Immobility in the FST according to stereotaxic injection with a Wnt signaling antagonist in naïve mice (each group N = 6). Data are presented as the means ± SE. * denote differences at *p* < 0.05.

A 4-week chronic mild stress protocol reduced the number of pGSK3β + cells and nuclear β-catenin levels, a reduction that was reversed by 4 weeks of treadmill running and creatine supplementation (Fig. 3A: for pGSK3β, F4, 25 = 23.97, *p* < 0.01; Fig. 2B: for nuclear β-catenin levels, F4, 15 = 10.67, *p* < 0.01). The microinjection of C59, a Wnt antagonist, into the DG enhanced immobility in the FST in naïve mice even 5 days after injection (Fig. 3C; t10 = -2.27, *p* < 0.05).

## DISCUSSION

The current study demonstrated that chronic mild stress-induced decrease of hippocampal neurogenesis was protected against by regular exercise and/or creatine supplementation through activation of the Wnt/GSK3β/β-catenin pathway in the hippocampal DG area.

First, we observed that chronic mild stress caused an increase in immobility in the TST and FST, which was reversed by regular exercise and/or creatine intake in our experimental paradigm. These results suggest the protective effects of regular exercise and creatine supplementation on chronic stress-induced depressive-like behaviors, which is a valid model for exploring mechanisms underlying this change. To elucidate the neurogenic effect of exercise and creatine in a chronic stressful state, we measured the number of Ki-67+ (a marker of proliferation) and DCX+ (a marker of differentiation) cells. The chronic stress-evoked decrease in the capacity of new-born cells to proliferate and differentiate into neuronal cells in the hippocampal DG was restored by exercise and/or creatine regimen, suggesting that regular exercise and/or creatine intake prevents the disruption of neurogenesis caused by chronic stress. This result corresponded well to our behavioral data that support that hippocampal neurogenesis is required for the behavioral effects of antidepressants, supporting our hypothesis^6-8^. Notably, the number of Ki-67+ and DCX+ cells of the mice that exercised and received creatine under stressful conditions was significantly than that in higher naïve mice, suggesting the greater antidepressant action of co-treatment. In fact, most antidepressant medications such as the selective serotonin reuptake inhibitors (SSRIs) enhance extracellular serotonin levels, while chronic stress reduces their levels^20^. Several studies have reported that exercise can enhance hippocampal neurogenesis through increased brain serotonin levels^21,22^. Furthermore, we previously revealed that a 4-week protocol of creatine supplementation enhanced the number of 5-hydroxytryptamine (5-HT)-positive cells in the dorsal raphe nuclei (where the serotonergic cell body is located) under chronic stressful stimuli, suggesting an increase in serotonin synthesis caused by creatine^18^. Based on the previous findings, exercise and/or creatine efficiently prevent chronic stress-elicited depressive phenotypes through serotonin-regulated improvement of hippocampal neurogenesis.

The neurogenic and serotonergic effects of exercise and/or creatine led us to further investigate the molecular mechanisms underlying this change and focus on the Wnt/GSK3β/β-catenin pathway. Wnt signaling, which has an oncogenic effect, is a conserved pathway in typical development. Wnt signaling is a key pathway that regulates GSK3β activity^23^. Wnt receptors, frizzled, and low-density lipoprotein receptor related protein (LRP) inhibit proteasomal-dependent degradation of β-catenin, resulting in the accumulation of β-catenin in the nucleus. β-catenin interacts with members of the T-cell factor/lymphoid enhancer factor (Tcf/LEF) family, and β-catenin-Tcf/LEF complex translocates into the nucleus where it exerts transcriptional regulation of target genes, including cyclin D1 and c-fos^24^. We found that chronic stress disrupted the Wnt/GSK3β/β-catenin pathway, while exercise and/or creatine treatment reversed the disruption in the hippocampal DG, indicating that chronic stress reduced the phosphorylation of GSK3β at the serine-9 residue and the translocation of β-catenin into nucleus, which was then reversed by exercise and/or creatine treatment. Interestingly, co-treatment had a greater effect. Much evidence has demonstrated that Wnt signaling plays a crucial role in the development of emotional- and mood-related illnesses such as depression and memory-related neurodegenerative pathways^10-12^. The phosphorylation and inhibitory activity of GSK3β is elevated by SSRI treatment through 5-HT1A receptors, an excitatory serotonin receptor subtype^25^. Furthermore, phosphorylated GSK3β was enhanced by ketamine or its antagonist, along with antidepressant effects^26,27^. With reference to hippocampal neurogenesis, Wnt3a- or Wnt2-responsive hippocampal neurogenesis was enhanced by SSRI treatment^11,12^. Furthermore, Wnt signaling-related key regulators, including Wnt3a, β-catenin, and cyclin D1, were profoundly augmented, along with the aberrant increase in hippocampal neurogenesis in a model of temporal lobe epilepsy^13,14^. These previously published findings at least partly support our results in this work. Finally, we conducted an experiment to examine the effects of local antagonism of Wnt signaling on psychomotor-related despair using stereotaxic microinjection of the Wnt antagonist, C59, into the hippocampal DG area in naïve mice to support our hypothesis concerning the direct relationship between Wnt signaling and the depressive phenotype. The immobility of mice was significantly enhanced by the injection with C59 5 days after treatment (by which time neurogenesis was different between the C59 and saline treatment groups in our preliminary tests, data not shown), suggesting that the disturbance of Wnt signaling in the hippocampal DG directly affects behavioral depression.

Collectively, chronic stress-induced defects of hippocampal neurogenesis were protected against by regular exercise and creatine supplementation via the canonical Wnt/GSK3β/β-catenin pathway, thereby overcoming the depressive phenotype.
